# The Response of Alpha‐Aminoadipic Acid (2‐AAA) to Short Term Lysine Ingestion in Healthy Individuals

**DOI:** 10.1002/edm2.70168

**Published:** 2026-02-21

**Authors:** E. Danielle Dean, Stacy Desine, Holly M. Smith, Amanda C. Doran, Jonathan D. Mosley, M. Wade Calcutt, Jane F. Ferguson

**Affiliations:** ^1^ Division of Diabetes, Endocrinology and Metabolism Vanderbilt University Medical Center Nashville Tennessee USA; ^2^ Department of Molecular Physiology and Biophysics Vanderbilt University Nashville Tennessee USA; ^3^ Division of Cardiovascular Medicine Vanderbilt University Medical Center Nashville Tennessee USA; ^4^ Division of Clinical Pharmacology Vanderbilt University Medical Center Nashville Tennessee USA; ^5^ Department of Biochemistry Mass Spectrometry Research Center, Vanderbilt University Nashville Tennessee USA

## Abstract

**Background:**

Higher circulating levels of the metabolite alpha‐aminoadipic acid (2‐AAA) associate with increased risk of diabetes and cardiometabolic disease. 2‐AAA is metabolised from lysine, an essential dietary amino acid. However, the effects of lysine intake on plasma levels of 2‐AAA were unclear. We measured post‐prandial changes in plasma and urine levels of 2‐AAA in healthy individuals in response to oral intake of ^13^C isotope‐labelled lysine and assessed relationships with markers of glucose homeostasis.

**Methods:**

We recruited healthy individuals (*N* = 16) to an acute lysine challenge. We administered 5 g of ^13^C Lysine‐HCL in 50 mL water as an oral bolus. We measured the appearance of ^13^C lysine and ^13^C 2‐AAA in plasma and urine over a period of 6 h post‐ingestion and assessed changes in insulin, C‐peptide, glucagon, and GLP‐1.

**Results:**

We found that ^13^C lysine and ^13^C 2‐AAA were detectable in plasma 30 min post‐ingestion, peaking on average 2 h post‐ingestion. Interestingly, non‐labelled lysine and non‐labelled 2‐AAA also increased. Individuals with higher plasma levels of ^13^C 2‐AAA post‐ingestion also had higher levels of ^13^C 2‐AAA in urine. The rate of appearance of 2‐AAA in plasma and excretion in urine differed between individuals and was associated with differences in waist‐to‐hip ratio (WHR). We observed increases in plasma insulin, C‐peptide, glucagon, and GLP‐1 post‐lysine ingestion.

**Conclusion:**

Our data suggest that orally ingested lysine is catabolised to 2‐AAA over several hours. However, lysine ingestion also stimulates an increase in 2‐AAA from endogenous sources. The rate of production and excretion differs between individuals, suggestive of controlled regulation of this metabolic pathway. Individuals with a higher WHR, indicative of greater visceral adiposity, may have increased excretion of 2‐AAA, tryptophan, and kynurenine.

## Graphical abstract

Higher circulating levels of the metabolite alpha‐aminoadipic acid (2‐AAA) associate with increased risk of diabetes and cardiometabolic disease. We found that administration of ^13^C lysine in healthy volunteers associates with inter‐individual variation in lysine‐2‐AAA metabolism and leads to increases in plasma insulin, C‐peptide, glucagon, and GLP‐1 post‐lysine ingestion.

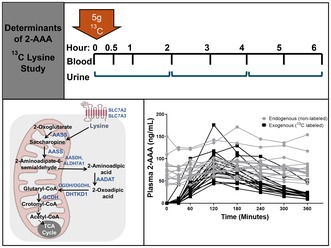

## 1 Introduction

Cardiometabolic diseases, including type 2 diabetes (T2D), cardiovascular disease (CVD), and related comorbidities, affect ~50% of adults worldwide [1]. Treatment of the known risk factors, including hypertension, hyperlipidemia, and smoking cessation, has led to significant improvements in morbidity and mortality [1]. However, these diseases continue to exert a significant burden, largely attributable to residual risk factors that remain to be fully elucidated, including inflammation and metabolic dysfunction [2]. Characterising these residual risk mechanisms has the potential to allow for significant additional improvement in prediction and treatment of cardiometabolic risk.

Plasma levels of the lysine‐derived metabolite α‐aminoadipic acid (2‐AAA) are associated with increased risk of type 2 diabetes [3], atherosclerosis [4], polycystic ovary syndrome [5], and cardiometabolic risk markers including low HDL cholesterol, obesity and higher liver fat [6, 7]. While these data establish 2‐AAA as an important biomarker of cardiometabolic disease risk, relatively little is known about the determinants of elevated 2‐AAA. Lysine is an exclusively ketogenic amino acid, and free lysine is transported into cells for catabolism through the saccharopine pathway in most tissues [8, 9, 10]. Following conversion of lysine to saccharopine, this is further converted to 2‐aminoadipate‐6‐semialdehyde, and then to 2‐AAA. 2‐AAA is then converted to 2‐oxoadipic acid, followed by breakdown to glutaryl CoA, crotonyl CoA, and ultimately to acetyl CoA [9]. An alternative pathway for production of 2‐AAA occurs through oxidation of protein‐bound lysine residues, mediated by myeloperoxidase (MPO) [11].

We have previously shown that changing the amount of lysine in the diet does not cause significant changes in fasting plasma 2‐AAA in healthy individuals [12]. However, the acute response of 2‐AAA to lysine in humans remains to be fully characterised. It has been found that there is increased conversion of lysine to 2‐AAA in individuals with high insulin, while the metabolic clearance rate of both lysine and 2‐AAA is altered in the setting of insulin resistance [13]. Glucagon signalling is also crucial for glucose homeostasis and interacts with amino acid signalling [14, 15]; however, the relationship between 2‐AAA and glucagon has not been studied. We administered an acute oral bolus of lysine (5 g ^13^C Lysine‐HCl), and measured levels of labelled and endogenous plasma and urine 2‐AAA serially over 6 h in healthy individuals, to understand how the availability of lysine affects levels of 2‐AAA post‐ingestion, and to probe potential relationships between 2‐AAA and mediators of glucose homeostasis.

## 2 Methods

### 2.1 Study Design

Healthy adult men and women (*N* = 17) were recruited to Vanderbilt University Medical Center between April 2022 and January 2023. Inclusion criteria included prior participation in the previous phases of the 2‐AAA Study (2‐AAA Cross‐sectional and 2‐AAA Lysine Intervention) [6, 12]. Exclusion criteria included pregnancy or lactation, newly diagnosed disease (cardiovascular, renal, liver, diabetes), inability to provide electronic informed consent, or inability to fast for 8 h. All participants provided written, informed consent, and the study was approved by the Vanderbilt University Institutional Review Board. The study was registered at ClinicalTrials.gov, NCT05210504. Study data were collected and managed using REDCap electronic data capture tools hosted at Vanderbilt University [16, 17].

### 2.2 Study Procedures

**FIGURE 1:**
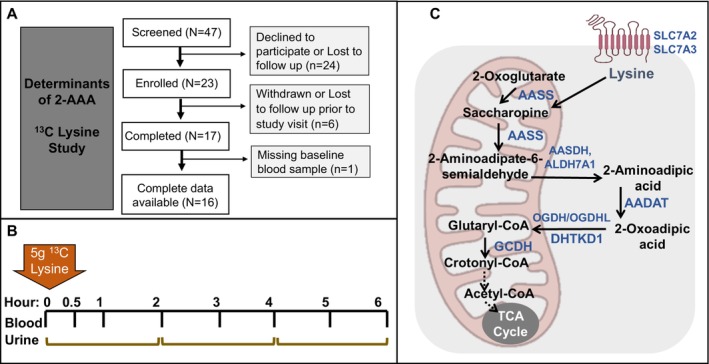
Design of 2‐AAA ^13^C lysine study and overview of lysine‐2‐AAA pathway. (A) Overview of screening and enrollment procedures. (B) Overview of study procedures and sample collection. (C) Overview of the lysine‐2‐AAA degradation pathway. We note that this pathway and its subcellular localization remain incompletely characterised, and it is not firmly established whether all steps take place within mitochondria or within both mitochondria and intra‐cellular space, as depicted.

An overview of the study is shown in Figure 1A,B. On the morning of the study visit, participants were asked to arrive ~8 AM, following an overnight fast (at least 8 h, no food or drinks other than water). Participants remained fasting throughout the entire study protocol, with no food or drink other than water. Vital signs were obtained, and a baseline urine sample was collected. An intravenous line was inserted into a superficial peripheral vein to perform blood draws, and a baseline blood sample was collected (5 mL EDTA tube). Immediately following the baseline blood sample, participants ingested 5 g ^13^C lysine (L‐Lysine·2HCl (^13^C_6_, 99%), Cambridge Isotope Laboratories), dissolved in 50 mL water. Whole blood (5 mL EDTA tube) was collected at serial times following lysine ingestion (30 min, 1 h, 2 h, 3 h, 4 h, 5 h, 6 h). Blood samples were immediately placed on ice and centrifuged (3000 ***g*** for 15 min at 4°C). Plasma aliquots were stored at −80°C prior to analysis. Total urine output during the 6‐h study visit was collected into three collection containers, from 0 to 2 h, 2–4 h and 4–6 h. One individual was missing a baseline blood sample and excluded from the analysis. Characteristics of the *N* = 16 individuals included in the analysis are shown in Table 1.

**TABLE 1:** Characteristics of the study participants of the 2‐AAA Lysine tracer study.

Participant characteristics	*N* or mean (SD)
Sex: male/female (*n*)	3/13
Race: White/Black/Asian (*n*)	14/1/1
Age (years) [mean (SD)]	33.5 (8.4)
BMI (kg/m^2^) [mean (SD)]	24.1 (3.1)
Waist to Hip ratio (WHR) [mean (SD)]	0.84 (0.07)
Blood pressure (mmHg) [mean (SD)]	112/74 (10/8)

### 2.3 Measurement of Metabolites and Proteins

Plasma and urine levels of lysine, 2‐AAA and related pathway metabolites (saccharopine, 2‐oxoadipic acid, Figure 1C) were quantified by liquid chromatography high‐resolution mass spectrometry (LC‐HRMS) at the Vanderbilt Mass Spectrometry Core. We also measured tryptophan and kynurenine, as control metabolites which are only distally related to the 2‐AAA pathway. Samples were spiked with internal standard (L‐tyrosine phenol‐3,5‐^13^C_2_, 95%–99%, Cambridge Isotopes, Andover, MA) and extracted with acetonitrile/methanol (2:1) containing 50 mM methoxyamine and 0.2% (v/v) TFA. Following centrifugation, the supernatants were collected and evaporated to dryness under a stream of nitrogen gas. In a separate glass tube, TFA anhydride was added dropwise to anhydrous isoamyl alcohol with vigorous stirring at 0°C to reach a final composition of 20% (v/v) anhydride, using extreme caution as appropriate when mixing anhydrides with alcohols, as these types of reactions can be explosive if not kept cold. Dried plasma or urine extracts were reconstituted in a solution of isoamyl alcohol/TFA anhydride, capped tightly, and incubated overnight at 50°C. The following day, samples were allowed to cool to room temperature, evaporated to dryness under a stream of nitrogen gas, and reconstituted in water/methanol (4:1) containing 0.1 M ammonium formate. The isoamyl methoxime derivatives of 2‐AAA ([M + H]^+^ 302.2326), 2‐AAA‐^13^C_6_ ([M + H]^+^ 308.2527), 2‐oxoAA ([M + H]^+^ 330.2275), 2‐oxoAA‐^13^C_6_ ([M + H]^+^ 336.2476), saccharopine ([M + H]^+^ 487.3742), saccharopine‐^13^C_6_ ([M + H]^+^ 493.3943), lysine ([M + H]^+^ 217.1911), lysine‐^13^C_6_ ([M + H]^+^ 223.2112), kynurenine ([M + H]^+^ 279.1703), kynurenine‐^13^C_6_ ([M + H]^+^ 285.1904), tryptophan ([M + H]^+^ 275.1754), and tryptophan‐^13^C_6_ ([M + H] + 281.1955) were measured by targeted selected ion monitoring (SIM) using a Vanquish ultrahigh performance liquid chromatography (UHPLC) system interfaced to a QExactive HF quadrupole/orbitrap mass spectrometer (Thermo Fisher Scientific). Data acquisition and quantitative spectral analysis were conducted using Thermo‐Finnigan Xcaliber version 4.1 and Thermo‐Finnigan LCQuan version 2.7, respectively. Calibration curves were constructed by plotting peak area ratios (analyte/internal standard) against analyte concentrations for a series of standards. Electrospray ionisation source parameters were tuned and optimised using an authentic 2‐AAA reference standard (Sigma Aldrich) derivatized with methoxyamine and isoamyl alcohol prior to direct liquid infusion. Samples were run in a single batch per tissue (plasma and urine) over a 48‐h period. Plasma levels of Insulin, C‐Peptide, Glucagon and GLP‐1 were measured in duplicate by ELISA (Mercodia) according to the manufacturer's instructions.

### 2.4 Statistical Methods

The change in plasma metabolites in response to ^13^C lysine ingestion was expressed as the area under the curve (AUC), calculated using the trapezoidal rule, adjusted for baseline levels. Urine metabolites were expressed as total amount excreted over the 6‐h period, calculated based on the total sum in nanograms (ng) for each collection period, adjusted for total urine volume. We assessed variables for skewness and kurtosis and determined that variables were normally distributed. Data were assessed for outliers (> ±3SD from the mean) and sensitivity analyses with or without inclusion of potential outliers performed to ensure that results were robust. Linear correlations between continuous variables were assessed by the Pearson correlation coefficient. Differences between individual timepoints were assessed using repeated‐measures ANOVA and paired‐sample *t*‐tests. *p* < 0.05 was considered statistically significant. Analyses were performed and results were visualised using Jamovi version 2.3.28 and GraphPad Prism version 9.4.1 (GraphPad Software, San Diego, CA).

## 3 Results

### 3.1 Ingestion of ^13^C Lysine Increases Circulating Lysine and 2‐AAA

**FIGURE 2:**
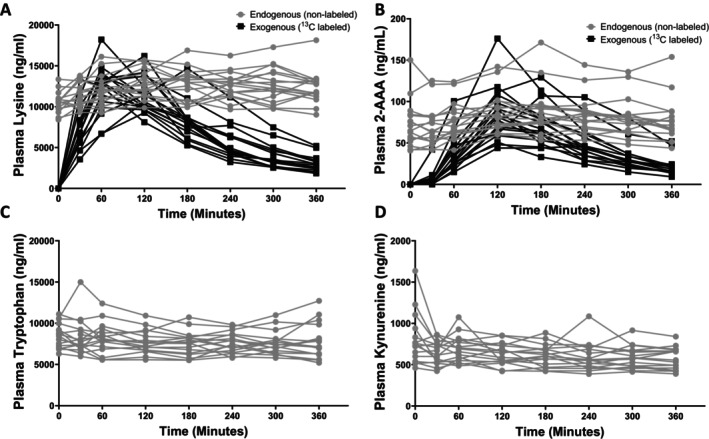
Acute ^13^C lysine supplementation caused a significant increase in plasma lysine and 2‐AAA, but not tryptophan or kynurenine. *p* < 0.05, Repeated‐measures ANOVA.

We observed significant increases over time in plasma levels of ^13^C lysine (*p* < 0.001, Figure 2A) and ^13^C 2‐AAA (*p* < 0.001, Figure 2B) following ingestion of the ^13^C lysine tracer. Notably, we also observed small but significant increases over time in plasma levels of endogenous (unlabeled) lysine (*p* < 0.001, Figure 2A) and unlabeled 2‐AAA (*p* = 0.005, Figure 2B). There was no significant change in plasma tryptophan (*p* = 0.478; Figure 2C) or kynurenine (*p* = 0.176; Figure 2D), suggesting that lysine ingestion specifically altered lysine‐related metabolism and did not affect amino acid metabolism more generally. We were unable to detect any ^13^C tryptophan or ^13^C kynurenine, suggesting that there was no conversion of ^13^C lysine to kynurenine through alternative catabolic pathways. We were unable to detect the lysine‐2‐AAA intermediates, saccharopine or 2‐oxoadipic acid in plasma, potentially due to extremely low levels or very short half‐life.

### 3.2 Urinary Excretion of Unlabeled and ^13^C‐Labelled Lysine and 2‐AAA Are Independently Correlated

**FIGURE 3:**
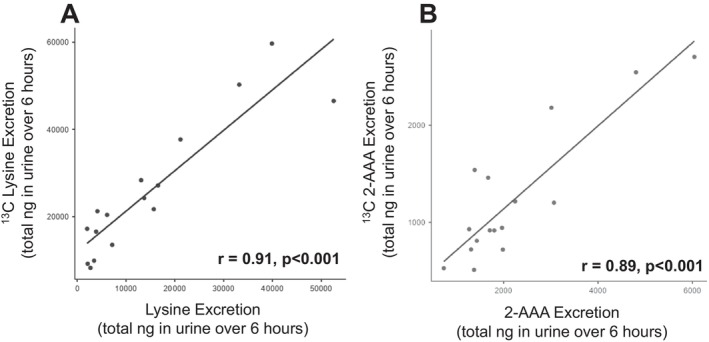
Urinary excretion of both ^13^C and unlabeled lysine (A) and 2‐AAA (B) is positively correlated.

We calculated the total amount of lysine and 2‐AAA excreted in urine over the 6‐h study period, based on the sum of the amount quantified within each 2‐h collection period, adjusted for total urine volume. We found that there was a significant positive correlation between the total amount of unlabeled lysine and ^13^C lysine excreted (*r* = 0.91, *p* < 0.001, Figure 3A), and between the total amount of unlabeled 2‐AAA and ^13^C 2‐AAA excreted (*r* = 0.89, *p* < 0.001, Figure 3B). There was a modest significant correlation between the amount of 2‐AAA excreted and the amount of lysine excreted when looking at the unlabeled (*r* = 0.524, *p* = 0.03) but not the ^13^C labelled measurements (*r* = 0.345, *p* = 0.2). We did not observe any differences by sex in the total amounts of lysine or 2‐AAA excreted; however, we note that our sample included only 3 men, limiting power to detect sex differences.

### 3.3 Higher Levels of Plasma ^13^C 2‐AAA Associate With Higher Levels of ^13^C 2‐AAA Excretion in Urine

**FIGURE 4:**
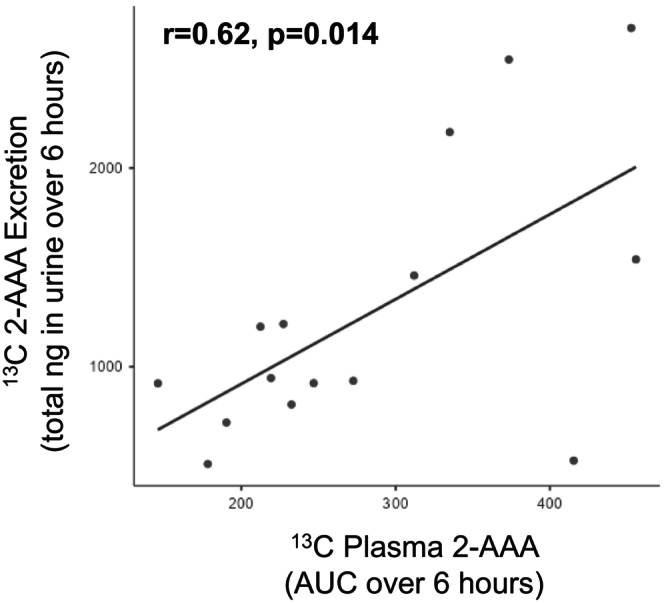
Levels of ^13^C 2‐AAA in plasma and urine are positively correlated following ^13^C lysine challenge. AUC calculated using the trapezoidal rule, adjusted for baseline levels.

We assessed the relationship between changes in plasma and urine levels of 2‐AAA. For plasma, we calculated the AUC over the 6‐h study period adjusted for baseline level, and compared it with total urinary 2‐AAA excreted over the study period. Our original hypothesis was that people with higher plasma 2‐AAA might have reduced urinary excretion. Our data showed that for the exogenously‐derived 2‐AAA, there was in fact a significant positive correlation between plasma and urine 2‐AAA (*r* = 0.62, *p* = 0.014, Figure 4), suggesting that people who metabolised more of the ^13^C lysine to ^13^C 2‐AAA in plasma also excreted more in urine. There is some evidence that high levels of lysine inhibit renal tubular protein reabsorption [18], raising the possibility that differences in circulating lysine may influence amino acid or metabolite reabsorption in the kidneys [19]. However, there was no significant relationship between the plasma AUC of lysine and urinary lysine excretion when looking at either unlabeled or ^13^C lysine levels.

### 3.4 The Rate of Production and Excretion of 2‐AAA Differs Between Individuals

All participants in the study received the same 5 g oral bolus of lysine; however, the rate of increase in plasma and amount excreted in urine varied considerably. Levels of ^13^C lysine in plasma, as calculated based on the AUC, ranged from 28,805 to 62,514 ng/mL/h, with a mean of 43,451 ng/mL/h. Total excretion of ^13^C lysine in urine ranged from 8258 to 59,679 ng, with a mean of 25,750 ng. Levels of ^13^C 2‐AAA in plasma, as calculated based on the AUC, ranged from 146 to 456 ng/mL/h, with a mean of 281 ng/mL/h. Total excretion of ^13^C 2‐AAA in urine ranged from 510 to 2706 ng, with a mean of 1240 ng. There was no association between either plasma or urine ^13^C lysine or 2‐AAA and sex or body weight; however, there was a negative association between plasma ^13^C lysine AUC and height, which remained significant when controlling for sex (*r* = −0.62, *p* = 0.01).

### 3.5 Plasma Levels of 2‐AAA Remain Stable Over Time

As the individuals participating in the lysine tracer study had previously completed a 2‐AAA cross‐sectional study [6] and a lysine dietary intervention study [12], we were able to compare their 2‐AAA levels against those measured previously (spanning an average time from first to last measurement of 2.5 years, ranging from a minimum interval of 11 months to a maximum interval of 3 years and 10 months). Plasma levels of 2‐AAA were stable over time, with a moderate‐high positive correlation between the first and last measurement (*r* = 0.66, *p* = 0.006), and no significant change over time (RM‐ANOVA *p* = 0.2).

### 3.6 The Change in 2‐AAA in Response to Lysine Associates With Greater Waist‐To‐Hip Ratio

Baseline plasma levels of unlabeled 2‐AAA were associated with greater waist‐to‐hip ratio (WHR; *r* = 0.62, *p* = 0.008). Similarly, the maximum levels of plasma 2‐AAA post‐ingestion also were associated with greater WHR (*r* = 0.64, *p* = 0.005). There was a trend in the same direction between WHR and plasma levels of ^13^C 2‐AAA, but this was not statistically significant. We found that greater urinary excretion of 2‐AAA was also associated with higher WHR when assessing either the labeled (*r* = 0.69, *p* = 0.004) or unlabeled measurements (*r* = 0.82, *p* < 0.001). Of note, there was also an association between WHR and urinary excretion of both tryptophan (*r* = 0.89, *p* < 0.001) and kynurenine (*r* = 0.93, *p* < 0.001), but not lysine. These associations remained significant in sex‐adjusted analyses.

### 3.7 Lysine Intake Induces Changes in Glucose‐Regulating Hormones

**FIGURE 5:**
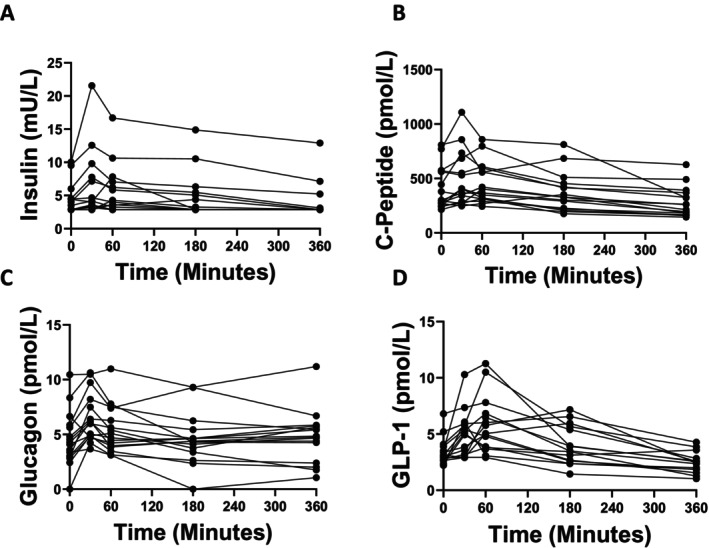
Glucose‐regulating hormones are increased following lysine ingestion. Insulin (A), C‐Peptide (B), Glucagon (C), and GLP‐1 (D) all changed significantly following lysine ingestion (all *p* < 0.001 by RM‐ANOVA). We assessed the impact of removing outliers (values > ±3 SD) and found consistent results, so we included all data for analysis/presentation.

As expected, lysine intake stimulated increases in glucose regulating hormones (Figure 5). Following ingestion of lysine, there was a significant increase in insulin (*p* < 0.001, Figure 5A), which peaked 60 min post‐ingestion and returned to basal levels by 3 h post‐ingestion. Similarly, C‐peptide levels changed significantly (*p* < 0.001, Figure 5B), with increased levels at 30 min (*p* < 0.05) before dipping significantly below baseline levels by 6 h post‐ingestion (*p* < 0.001). Levels of glucagon increased 30 min post‐ingestion (p < 0.001, Figure 5C), remaining elevated at 60 min (*p* = 0.04) before returning to basal levels at the 3 and 6‐h timepoints. Levels of GLP‐1 changed significantly (*p* < 0.001, Figure 5D), with significant increases at 30 (*p* = 0.01) and 60 min (p < 0.001) before falling below baseline levels by 6‐h post‐ingestion (*p* = 0.002). Higher baseline levels of 2‐AAA associated with a greater insulin response (defined as the AUC adjusted for baseline), *p* = 0.01.

## 4 Discussion

There is strong interest in the metabolite 2‐AAA as a potential biomarker of cardiometabolic disease; however, the determinants of 2‐AAA remain poorly characterised. 2‐AAA is derived from the catabolism of the essential amino acid lysine in mitochondria or peroxisomes, through the saccharopine or pipecolic acid pathways respectively [8, 9, 10]. Whether dietary lysine is the predominant determinant of circulating levels of 2‐AAA was not known. We previously found that 1 week of dietary lysine supplementation did not significantly alter fasting levels of plasma 2‐AAA [12]. However, it was not known whether ingestion of lysine associates with altered post‐prandial levels of 2‐AAA. We examined this question in the current study and found that an oral bolus of ^13^C lysine led to increased levels of ^13^C 2‐AAA in the hours following ingestion. Strikingly, there were also increases in endogenously‐produced 2‐AAA, suggesting that lysine intake may stimulate increases in 2‐AAA independent of its role as a 2‐AAA substrate. There was variability in the 2‐AAA response to lysine, which associated with differences in WHR, suggestive of controlled regulation of this pathway which may be altered in the setting of high central adiposity. This further supports our hypothesis that 2‐AAA is an important signalling molecule in nutrient and metabolic homeostasis, which is under complex control and has implications for cardiometabolic pathophysiology.

We hypothesized that levels of 2‐AAA are influenced both by direct catabolism of free lysine and by production of 2‐AAA from stored lysine or from catabolism of protein‐bound lysine residues, and that this is under metabolic control which differs between individuals. Our data support this hypothesis: while plasma and urine 2‐AAA increased in response to the lysine bolus in all individuals, there was significant inter‐individual variability in the magnitude of these increases. Notably, we found significant increases in non‐labelled 2‐AAA and lysine following the lysine bolus, which suggests that even in the setting of high free lysine availability, lysine or its downstream products may stimulate additional production or secretion of 2‐AAA and lysine. This might suggest that individuals secreted endogenous lysine and 2‐AAA in response to lysine ingestion. However, given the large amount of exogenous lysine provided, it is also possible that the influx of ^13^C lysine and ^13^C 2‐AAA competed with endogenous lysine and 2‐AAA for degradation, leading to the apparent increase in endogenous lysine and 2‐AAA.

Levels of 2‐AAA in both plasma and urine were positively correlated with each other, disproving the hypothesis that elevated plasma 2‐AAA are linked to lower urinary clearance of 2‐AAA. Further, there was no significant association between plasma or urine levels of 2‐AAA and lysine, which suggests that higher levels of 2‐AAA are not related to increased lysine catabolism. We thus speculate that higher 2‐AAA results from decreased downstream catabolism, potentially as part of a tightly controlled process that may regulate metabolism and oxidative stress, and may be disrupted in disease settings [20, 21, 22, 23]. We attempted to quantify levels of other lysine‐2‐AAA pathway metabolites in plasma, including saccharopine and 2‐oxoadipic acid; however, the levels were not detectable, likely due to the short half‐life of these intermediate metabolites. Saccharopine accumulation has been shown to be toxic to mitochondria [24], and is likely rapidly catabolised in healthy individuals. Future in‐depth mechanistic studies in animal models may be required to further probe tissue‐specific catabolism of 2‐AAA.

The amount of ^13^C lysine and ^13^C 2‐AAA detected in plasma and urine varied considerably. We observed a negative association between plasma ^13^C lysine AUC and height; this might be attributable to physiological differences related to the determination of height during early life [25], however, we caution that this finding requires replication. Differences in ^13^C lysine and ^13^C 2‐AAA may be attributable to varying rates of intestinal absorption of lysine. It is also possible that direct metabolism of lysine by gut microbiota contributes to this variability [26, 27]. We note that we did not collect stool samples from these individuals, and thus are unable to resolve whether differences in plasma and urine lysine and 2‐AAA relate to differences in faecal excretion of lysine and 2‐AAA. We previously found that plasma 2‐AAA associates with dietary consumption of animal products [12], while plasma levels of 2‐AAA have also been found to associate with a high sodium diet [28]. Further studies are needed to examine the interplay of diet and microbiota in 2‐AAA homeostasis.

Fasting levels of plasma 2‐AAA were relatively stable over time, with high correlation between levels in samples taken several years apart. This is consistent with published studies [29, 30, 31]. A previous study found that plasma levels of 2‐AAA showed evidence of circadian rhythmicity in normal weight and obese individuals, but not in individuals with T2D [32]. We did not include a placebo group in our study, and cannot exclude the possibility that our observed increases in endogenous 2‐AAA may have been attributable to circadian effects rather than the lysine ingestion. However, we note that in the previous study, 2‐AAA peaked in late afternoon rather than the morning and also included meals, and as such the changes observed in 2‐AAA could have been due to post‐prandial effects rather than circadian effects per se. Further studies are required to fully characterise the response of 2‐AAA to different dietary and other stimuli.

Previous studies in humans have helped to further characterise the relationship between insulin and 2‐AAA. High levels of insulin, induced by euglycemic clamp, increased lysine to 2‐AAA conversion in both lean and overweight women and increased clearance of 2‐AAA in the overweight insulin‐resistant women, but not lean control women [13]. High levels of glucagon in the setting of insulin deficiency were shown to increase 2‐AAA through increased lysine to 2‐AAA conversion in the splanchnic bed [33]. Here we found significant increases in insulin, glucagon, C‐peptide, and GLP‐1 in response to lysine ingestion. Recent studies from our group have shown the lysine transporter *SLC7A2* is highly expressed in liver as well as pancreatic islet alpha and beta cells secreting glucagon and insulin, respectively [34, 35] suggesting a potential mechanism for lysine's effects on islet hormone secretion. Further, higher baseline 2‐AAA was associated with a greater insulin response, which is consistent with data in mice, which found that supplementation with 2‐AAA caused higher insulin secretion [3].

This study has considerable strengths, including the use of ^13^C isotope labelled lysine to examine 2‐AAA production, and the use of a well‐phenotyped human longitudinal sample. However, there are also some limitations, including relatively few male participants and technical limitations in quantifying all intermediate metabolites in the lysine‐2‐AAA pathway. We provided a large bolus of ^13^C lysine, which allowed us to examine its appearance and catabolism to 2‐AAA, but this may also have perturbed endogenous lysine metabolism. We measured tryptophan as a control amino acid that can be both ketogenic and glucogenic and found no significant change, but cannot exclude potential changes in other amino acids. We measured metabolites in plasma and urine; these are easily accessible tissues, with clinical relevance; however, these may not reflect metabolism of 2‐AAA in tissues. Future studies using animal and cell models are required to further probe mechanisms.

In summary, our study demonstrates that ingestion of lysine causes a transient increase in levels of 2‐AAA, but that the 2‐AAA response differs between individuals. Individuals with high plasma 2‐AAA also have higher urine levels of 2‐AAA, suggesting that elevated 2‐AAA is due to decreased catabolism of 2‐AAA rather than differences in direct urinary excretion. We found that individuals with higher WHR, indicative of central adiposity, had higher levels of 2‐AAA. However, further studies are needed to fully define the mechanisms underlying elevated 2‐AAA.

## Author Contributions

Conceptualization (J.F.F., E.D.D., A.C.D.), Data curation (S.D., H.M.S., M.W.C., J.F.F.), Funding acquisition (J.F.F., E.D.D.), Investigation (S.D., H.M.S., J.D.M., M.W.C.), Writing – original draft (J.F.F.), Writing – review and editing (E.D.D., S.D., H.M.S., A.C.D., J.D.M., M.W.C.).

## Funding

This study was funded by the National Institute of Diabetes and Digestive and Kidney Diseases (R01 DK117144 to J.F.F.). The project was also supported by funding from the American Heart Association (24TPA1278431 to J.F.F.) and the Vanderbilt Institute for Clinical and Translational Research (VICTR) through CTSA award UL1TR002243 from the National Center for Advancing Translational Sciences (NCATS). The REDCap project was funded by UL1TR000445 from NCATS/NIH. The contents of this study are solely the responsibility of the authors and do not necessarily represent official views of the National Institutes of Health.

## Conflicts of Interest

The authors declare no conflicts of interest.

## Data Availability

The data that support the findings of this study are available from the corresponding author upon reasonable request.

## References

[1] S. S. Martin , A. W. Aday , Z. I. Almarzooq , et al., “2024 Heart Disease and Stroke Statistics: A Report of US and Global Data From the American Heart Association,” Circulation 149 (2024): e347–e913, 10.1161/CIR.0000000000001209. 38264914 PMC12146881

[2] A. W. Aday and P. M. Ridker , “Targeting Residual Inflammatory Risk: A Shifting Paradigm for Atherosclerotic Disease,” Frontiers in Cardiovascular Medicine 6 (2019): 16, 10.3389/fcvm.2019.00016. 30873416 PMC6403155

[3] T. J. Wang , D. Ngo , N. Psychogios , et al., “2‐Aminoadipic Acid Is a Biomarker for Diabetes Risk,” Journal of Clinical Investigation 123 (2013): 4309–4317, 10.1172/JCI64801. 24091325 PMC3784523

[4] A. Saremi , S. Howell , D. C. Schwenke , et al., “Advanced Glycation End Products, Oxidation Products, and the Extent of Atherosclerosis During the VA Diabetes Trial and Follow‐Up Study,” Diabetes Care 40 (2017): 591–598, 10.2337/dc16-1875. 28148544 PMC5360279

[5] A. Y. Chang , A. Z. Lalia , G. D. Jenkins , et al., “Combining a Nontargeted and Targeted Metabolomics Approach to Identify Metabolic Pathways Significantly Altered in Polycystic Ovary Syndrome,” Metabolism 71 (2017): 52–63, 10.1016/j.metabol.2017.03.002. 28521878 PMC5520539

[6] S. Desine , C. L. Gabriel , H. M. Smith , et al., “Association of Alpha‐Aminoadipic Acid With Cardiometabolic Risk Factors in Healthy and High‐Risk Individuals,” Frontiers in Endocrinology 14 (2023): 1122391. 37745703 10.3389/fendo.2023.1122391 PMC10513411

[7] M. Shi , C. Wang , H. Mei , et al., “Genetic Architecture of Plasma Alpha‐Aminoadipic Acid Reveals a Relationship With High‐Density Lipoprotein Cholesterol,” Journal of the American Heart Association 11 (2022): e024388, 10.1161/JAHA.121.024388. 35621206 PMC9238724

[8] Y. F. Chang , “Pipecolic Acid Pathway: The Major Lysine Metabolic Route in the Rat Brain,” Biochemical and Biophysical Research Communications 69 (1976): 174–180. 1259753 10.1016/s0006-291x(76)80288-4

[9] I. A. Pena , L. A. Marques , A. B. Laranjeira , et al., “Mouse Lysine Catabolism to Aminoadipate Occurs Primarily Through the Saccharopine Pathway; Implications for Pyridoxine Dependent Epilepsy (PDE),” Biochimica et Biophysica Acta 1863 (2017): 121–128, 10.1016/j.bbadis.2016.09.006. 27615426

[10] D. E. Matthews , “Review of Lysine Metabolism With a Focus on Humans,” Journal of Nutrition 150 (2020): 2548S–2555S, 10.1093/jn/nxaa224. 33000162

[11] H. Lin , B. S. Levison , J. A. Buffa , et al., “Myeloperoxidase‐Mediated Protein Lysine Oxidation Generates 2‐Aminoadipic Acid and Lysine Nitrile In Vivo,” Free Radical Biology & Medicine 104 (2017): 20–31, 10.1016/j.freeradbiomed.2017.01.006. 28069522 PMC5353359

[12] O. R. Antonetti , S. Desine , H. M. Smith , et al., “The Consumption of Animal Products Is Associated With Plasma Levels of Alpha‐Aminoadipic Acid (2‐AAA),” Nutrition, Metabolism, and Cardiovascular Diseases 34 (2024): 1712–1720, 10.1016/j.numecd.2024.03.009. PMC11188583 38658223

[13] A. Y. Chang , A. K. Asokan , A. Z. Lalia , et al., “Insulin Regulation of Lysine and α‐Aminoadipic Acid Dynamics and Amino Metabolites in Women With and Without Insulin Resistance,” Diabetes 73 (2024): 1592–1604, 10.2337/db23-0977. 38968429 PMC11417443

[14] E. D. Dean , “A Primary Role for α‐Cells as Amino Acid Sensors,” Diabetes 69 (2020): 542–549, 10.2337/dbi19-0021. 31653720 PMC7085241

[15] S. L. Armour , J. E. Stanley , J. Cantley , E. D. Dean , and J. G. Knudsen , “Metabolic Regulation of Glucagon Secretion,” Journal of Endocrinology 259 (2023): e230081, 10.1530/JOE-23-0081. 37523232 PMC10681275

[16] P. A. Harris , R. Taylor , R. Thielke , J. Payne , N. Gonzalez , and J. G. Conde , “Research Electronic Data Capture (REDCap)—A Metadata‐Driven Methodology and Workflow Process for Providing Translational Research Informatics Support,” Journal of Biomedical Informatics 42 (2009): 377–381, 10.1016/j.jbi.2008.08.010. 18929686 PMC2700030

[17] P. A. Harris , R. Taylor , B. L. Minor , et al., “The REDCap Consortium: Building an International Community of Software Platform Partners,” Journal of Biomedical Informatics 95 (2019): 103208, 10.1016/j.jbi.2019.103208. 31078660 PMC7254481

[18] C. E. Mogensen and K. Sølling , “Studies on Renal Tubular Protein Reabsorption: Partial and Near Complete Inhibition by Certain Amino Acids,” Scandinavian Journal of Clinical and Laboratory Investigation 37 (1977): 477–486, 10.3109/00365517709101835. 98829

[19] F. Verrey , D. Singer , T. Ramadan , R. N. Vuille‐dit‐Bille , L. Mariotta , and S. M. R. Camargo , “Kidney Amino Acid Transport,” Pflügers Archiv / European Journal of Physiology 458 (2009): 53–60, 10.1007/s00424-009-0638-2. 19184091

[20] F. L. Theodoulou , O. C. M. Sibon , S. Jackowski , and I. Gout , “Coenzyme A and Its Derivatives: Renaissance of a Textbook Classic,” Biochemical Society Transactions 42 (2014): 1025–1032, 10.1042/BST20140176. 25109997

[21] L. Shi and B. P. Tu , “Acetyl‐CoA and the Regulation of Metabolism: Mechanisms and Consequences,” Current Opinion in Cell Biology 33 (2015): 125–131, 10.1016/j.ceb.2015.02.003. 25703630 PMC4380630

[22] K. E. Wellen and C. B. Thompson , “A Two‐Way Street: Reciprocal Regulation of Metabolism and Signalling,” Nature Reviews. Molecular Cell Biology 13 (2012): 270–276, 10.1038/nrm3305. 22395772

[23] V. Filonenko and I. Gout , “Discovery and Functional Characterisation of Protein CoAlation and the Antioxidant Function of Coenzyme A,” BBA Advances 3 (2023): 100075, 10.1016/j.bbadva.2023.100075. 37082257 PMC10074942

[24] J. Zhou , X. Wang , M. Wang , et al., “The Lysine Catabolite Saccharopine Impairs Development by Disrupting Mitochondrial Homeostasis,” Journal of Cell Biology 218 (2019): 580–597, 10.1083/jcb.201807204. 30573525 PMC6363459

[25] M. Shi , A. M. Manouchehri , C. M. Shaffer , et al., “Genetic Thyrotropin Regulation of Atrial Fibrillation Risk Is Mediated Through an Effect on Height,” Journal of Clinical Endocrinology and Metabolism 106 (2021): 2124–2132, 10.1210/clinem/dgab272. 33895829 PMC8208678

[26] C. Esmahan , E. Alvarez , E. Montenegro , and J. F. Martin , “Catabolism of Lysine in Penicillium Chrysogenum Leads to Formation of 2‐Aminoadipic Acid, a Precursor of Penicillin Biosynthesis,” Applied and Environmental Microbiology 60 (1994): 1705–1710, 10.1128/aem.60.6.1705-1710.1994. 8031073 PMC201551

[27] C. C. Metges , A. E. El‐Khoury , L. Henneman , et al., “Availability of Intestinal Microbial Lysine for Whole Body Lysine Homeostasis in Human Subjects,” American Journal of Physiology 277 (1999): E597–E607. 10516118 10.1152/ajpendo.1999.277.4.E597

[28] R. Hamaya , Q. Sun , J. Li , et al., “24‐h Urinary Sodium and Potassium Excretions, Plasma Metabolomic Profiles, and Cardiometabolic Biomarkers in the United States Adults: A Cross‐Sectional Study,” American Journal of Clinical Nutrition 120 (2024): 153–161, 10.1016/j.ajcnut.2024.05.010. 38762185 PMC11251214

[29] M. Breier , S. Wahl , C. Prehn , et al., “Targeted Metabolomics Identifies Reliable and Stable Metabolites in Human Serum and Plasma Samples,” PLoS One 9 (2014): e89728, 10.1371/journal.pone.0089728. 24586991 PMC3933650

[30] M. Carayol , I. Licaj , D. Achaintre , et al., “Reliability of Serum Metabolites Over a Two‐Year Period: A Targeted Metabolomic Approach in Fasting and Non‐Fasting Samples From EPIC,” PLoS One 10 (2015): e0135437, 10.1371/journal.pone.0135437. 26274920 PMC4537119

[31] T. Long , M. Hicks , H. C. Yu , et al., “Whole‐Genome Sequencing Identifies Common‐To‐Rare Variants Associated With Human Blood Metabolites,” Nature Genetics 49 (2017): 568–578, 10.1038/ng.3809. 28263315

[32] C. M. Isherwood , D. R. Van der Veen , J. D. Johnston , and D. J. Skene , “Twenty‐Four‐Hour Rhythmicity of Circulating Metabolites: Effect of Body Mass and Type 2 Diabetes,” FASEB Journal 31 (2017): 5557–5567, 10.1096/fj.201700323R. 28821636 PMC5690388

[33] H. James , W. I. Gonsalves , S. Manjunatha , et al., “The Effect of Glucagon on Protein Catabolism During Insulin Deficiency: Exchange of Amino Acids Across Skeletal Muscle and the Splanchnic Bed,” Diabetes 71 (2022): 1636–1648, 10.2337/db22-0079. 35621914 PMC9490357

[34] E. Spears , J. E. Stanley , M. Shou , et al., “Pancreatic Islet α Cell Function and Proliferation Requires the Arginine Transporter SLC7A2,” (2023), 10.1101/2023.08.10.552656. PMC13262736 42294887

[35] K. C. Coate , C. Dai , A. Singh , et al., “Interruption of Glucagon Signaling Augments Islet Non‐Alpha Cell Proliferation in SLC7A2‐ and mTOR‐Dependent Manners,” Molecular Metabolism 90 (2024): 102050, 10.1016/j.molmet.2024.102050. 39433176 PMC11570739

